# Case Report: Stroke Chameleon: Acute Large Vessel Occlusion in the Posterior Circulation With Paroxysmal Sympathetic Hyperactivity as the First Manifestation

**DOI:** 10.3389/fnins.2022.890678

**Published:** 2022-05-24

**Authors:** Juntao Yin, Wan Wang, Yu Wang, Guofeng Li, Yongmei Kong, Xiaoqiang Li, Yingdong Xu, Yuqing Wei

**Affiliations:** ^1^Department of Neurology, Xingtai Third Hospital, Xingtai, China; ^2^Department of Neurology, Xingtai People's Hospital, Xingtai, China

**Keywords:** paroxysmal sympathetic hyperactivity, acute large vessel occlusion, posterior circulation, endovascular treatment, multiple lesions

## Abstract

Paroxysmal sympathetic hyperactivity (PSH) is a neurological emergency mostly secondary to traumatic brain injury (TBI). Acute large vessel occlusion (LVO) in the posterior circulation with PSH as the initial manifestation is uncommon. It may lead to catastrophic consequences for patients if not detected and treated timely. Here, we present three patients with acute LVO in the posterior circulation with PSH as the initial symptom. All patients were male and averaged 63 years old. The PSH Assessment Measure (PSH-AM) scores of all cases were > 17. Brain imaging showed that multiple lesions in posterior circulation were involved in three patients. Although the prognosis of all patients was poor, PSH symptoms disappeared in all patients after endovascular treatment. These cases suggests that acute posterior circulation-related ischemic stroke should be considered with PSH occurring as the first symptom. Extensive disconnection due to multiple lesions in posterior circulation may play an important role in the occurrence and development of PSH. Endovascular treatment may be effective for PSH caused by acute posterior circulation-related ischemic stroke. This is worthy of further study in the future.

## Introduction

Paroxysmal sympathetic hyperactivity (PSH) is mainly reported in traumatic brain injury (TBI) cases. It includes striking clinical features of exacerbated sympathetic activity, including excessive sweating, tachycardia, arterial hypertension, tachypnoea, hyperthermia, decorticate or decerebrate posturing and increased muscle tone (Meyfroidt et al., 2017). PSH is often misdiagnosed as seizure, severe sepsis or bacteremia due to the lack of clinical experience (Godoy et al., 2019), especially as the first manifestation of some diseases. As a devastating stroke, acute large vessel occlusion (LVO) in the posterior circulation often leads to a very poor clinical prognosis. However, acute ischemic stroke is treatable if diagnosed early. To the best of our knowledge, there have been no reports describing PSH as the first manifestation of acute ischemic stroke. Here, we report three cases of acute LVO in the posterior circulation stroke with PSH as the first clinical manifestation and summarize the relevant literature in order to improve the understanding of this phenomenon, particularly emergency physicians.

## Case Presentation

The cases are summarized in Table 1. All patients were male, with a mean age of 63 (range, 61–65) years. Mean Glasgow Coma Scale (GCS) and PSH Assessment Measure (PSH-AM) scores were 4 and 19 (range, 18–20) (Baguley et al., 2014), respectively. All cases were successfully recanalized (TICI grade III) after mechanical thrombectomy, and the average time from symptom onset to recanalization was 358 min (range, 175–480). Two of the three patients died during the 90-day follow up period, and the mRS score of the survivor was 4.

**Table 1:** Patient demographics and procedural details.

**Factor**	**Case 1**	**Case 2**	**Case 3**
Gender	Male	Male	Male
Age (years)	64	61	65
Vascular risk factors	Hypertension, type 2 DM and PAF	Hypertension, type 2 DM and SAF	Hypertension and SAF
GCS score	4	4	4
PSH-AM score	18	19	20
Type of EVT	Mechanical thrombectomy	Mechanical thrombectomy	Mechanical thrombectomy
Time from onset to recanalization (min)	420	175	480
Outcome of EVT (TICI grade)	III	III	III
Infarct site	Thalamus, corpus callosum, occipital lobe, temporal lobe, cerebellar hemisphere, cerebellar vermis, midbrain and pons	Bilateral thalamus, midbrain, occipital lobe, cerebellar vermis and pons	Bilateral cerebellar hemispheres, cerebellar vermis and PAG
Admission length	3 weeks	5 months	10 hours
mRS at 90-day follow-up	Dead	5	Dead

*GCS, Glasgow Coma Scale; PSH-AM, paroxysmal sympathetic hyperactivity assessment measure; EVT, endovascular treatment; DM, diabetes mellitus; PAF, paroxysmal atrial fibrillation; SAF, sustained atrial fibrillation, PAG, periaqueductal gray*.

### Case 1

**Figure 1:**
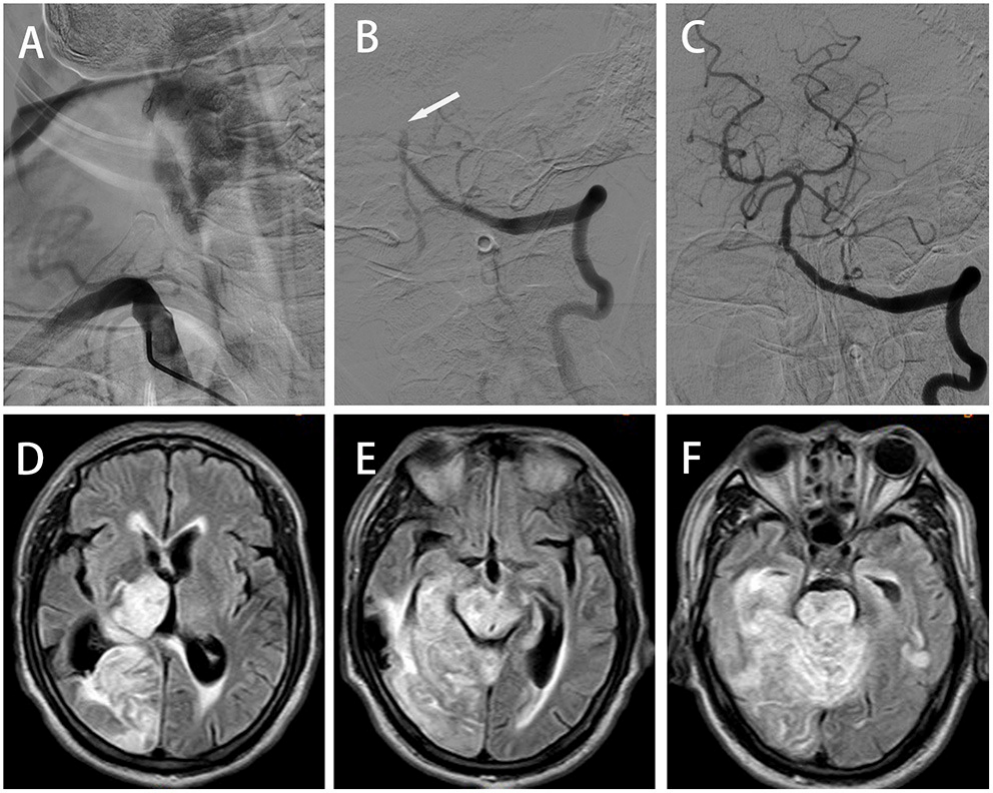
Case 1: **(A)** Anterior–posterior (AP) digital subtraction angiography (DSA) showing occlusion at the beginning of the right vertebral artery (VA). AP left VA angiogram demonstrating basilar artery occlusion (**(B)**, arrowheads); successful recanalization was obtained by endovascular thrombectomy **(C)**. **(D–F)** Axial FLAIR magnetic resonance imagining (MRI) showing multiple infarction lesions, involving the right thalamus, corpus callosum, right occipital lobe, right temporal lobe, right cerebellar hemisphere, cerebellar vermis, midbrain and pons.

A 64-year-old male patient was admitted to our hospital for vomiting and limb shaking for 4 h and unconsciousness for 3 h. He had a past medical history of hypertension and type 2 diabetes mellitus (DM). He had cerebral infarction 12 years ago with mild residual weakness on the right side. On admission physical examination revealed coma with tetraplegia, paroxysmal limb shaking (spontaneous symptoms lasting an average of 30 s at an average interval of 20 min), bilateral and spontaneous extensor posturing, unequal pupils (left, 2 mm; right, 4 mm) with sluggishness to light, bilateral positive Babinski's sign, excessive sweating, fever (38.0°C), tachycardia (102/min), tachypnea (22/min) and hypertension (180/87 mmHg). His GCS score was 4. The above manifestations led to the diagnosis of PSH (PSH-AM score of 18). Brain computed tomography (CT) scan was normal except for chronic infarction in the temporal and occipital lobes. His blood biochemistry, complete blood count and chest CT scan were normal. His electroencephalogram (EEG) showed no epileptic waves. Acute posterior circulation-associated ischemic stroke was considered. The patient was not administered recombinant human tissue plasminogen activator (rt-PA) due to concurrent stress ulceration. Digital subtraction angiography (DSA) demonstrated right vertebral artery (VA) and basilar artery occlusion (Figures 1A,B). Subsequently, the patient underwent mechanical thrombectomy using retrievable stent devices, and the basilar artery was successfully recanalized (TICI grade III, Figure 1C). The time from symptom onset to recanalization was 420 min. Blood pressure of the patient fluctuated around 150/100 mmHg and body temperature returned to normal within 10 h after the operation, and symptoms of sweating, tachypnea and paroxysmal limb shaking did not occur again. However, the patient remained in a coma and was transferred to the neurological intensive care unit (NICU). At postoperative day 5, brain magnetic resonance imaging (MRI) revealed multiple infarcts, involving the right thalamus, corpus callosum, right occipital lobe, right temporal lobe, right cerebellar hemisphere, cerebellar vermis, midbrain and pons (Figures 1D–F). During this period, paroxysmal atrial fibrillation was detected by 24-h dynamic electrocardiography. Unfortunately, the patient condition did not improve after 3 weeks in the NICU, and he was still in a vegetative state at discharge. PSH symptoms did not recur during the rehabilitation phase. The patient died of lung infection 3 months after discharge.

### Case 2

**Figure 2:**
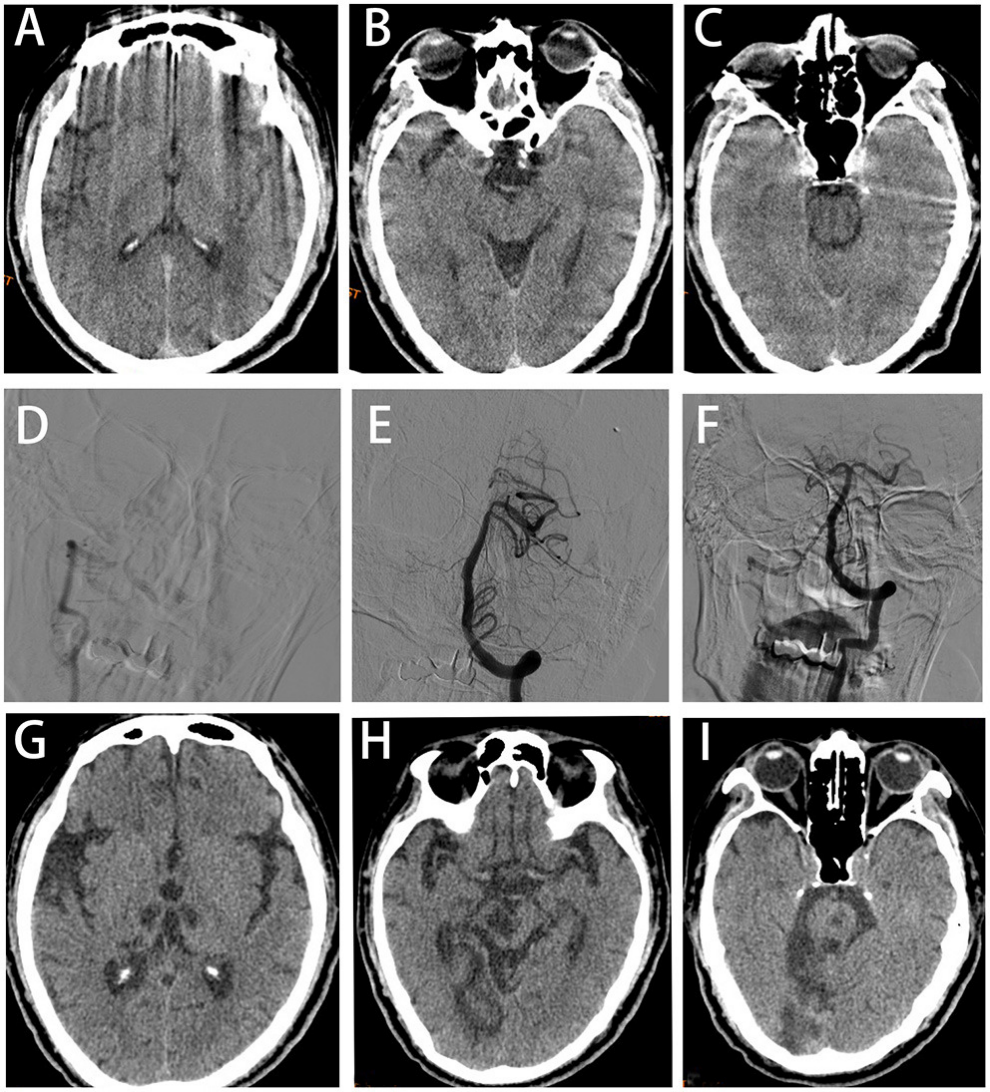
Case 2: **(A–C)** Axial brain computed tomography (CT) scan revealing no abnormalities. **(D)** AP right VA angiogram showing V4 segment occlusion. **(E)** AP left VA angiogram showing right superior cerebellar artery (SCA) and right posterior cerebral artery (PCA) occlusions; these vessels were successfully recanalized by endovascular thrombectomy **(F)**. **(G–I)** Axial brain CT at 3 postoperative months showing low-density lesions in the bilateral thalamus, midbrain, right occipital lobe, cerebellar vermis and pons.

A 61-year-old male was admitted in the emergency department of our hospital for 1 h due to sudden loss of consciousness. He had a history of cerebral infarction, hypertension, type 2 DM, and atrial fibrillation. He was in a deep coma (GCS score of 4) with some striking signs such as excessive sweating, decerebrate rigidity, paroxysmal limb shaking (spontaneous symptoms lasting an average of 10 s at an average interval of 30 min), fever (41°C), tachycardia (112/min), hypertension (165/95 mmHg), tachypnea (25/min) and bilateral positive Babinski's sign. PSH was diagnosed based on a PSH-AM score of 19. CT of the head (Figures 2A–C) and chest showed no abnormality. ECG revealed persistent atrial fibrillation and EEG showed no epileptic waves. Laboratory parameters were normal except white blood cell (WBC) count (15.44 × 10^9^/L) and neutrophil count (13.44 × 10^9^/L). The standard dose of rt-PA was injected intravenously for acute ischemic stroke. However, the clinical symptom did not improve. DSA revealed the V4 segment of the right VA (Figure 2D), right superior cerebellar artery (SCA) and P1 segment of the right posterior cerebral artery (PCA) were occluded (Figure 2E). The right SCA and right PCA were recanalized after mechanical thrombectomy (TICI grade III, Figure 2F). The time from symptom onset to recanalization was 175 min. The patient was still in coma with tetraplegia after surgery. Subsequently he was transferred to the NICU and received treatment of atorvastatin, edaravone, omeprazole, ambroxol hydrochloride and life-sustaining treatments. After the vital signs were stable, he was transferred 44 days postoperatively to our rehabilitation center. Brain CT scan at 3 months after the operation showed that low-density lesions in the bilateral thalami, midbrain, right occipital lobe, cerebellar vermis and pons (Figures 2G–I). The patient's condition improved (GCS score of 11), and he was discharged after 5 months of hospitalization (mRS score of 5). During the whole rehabilitation period, there was no recurrence of sweating, tachypnea and paroxysmal limb shaking. The patient left quadriplegia, unclear speech and dysphagia and was hospitalized repeatedly due to pulmonary infection during a 3-year follow-up period.

### Case 3

**Figure 3:**
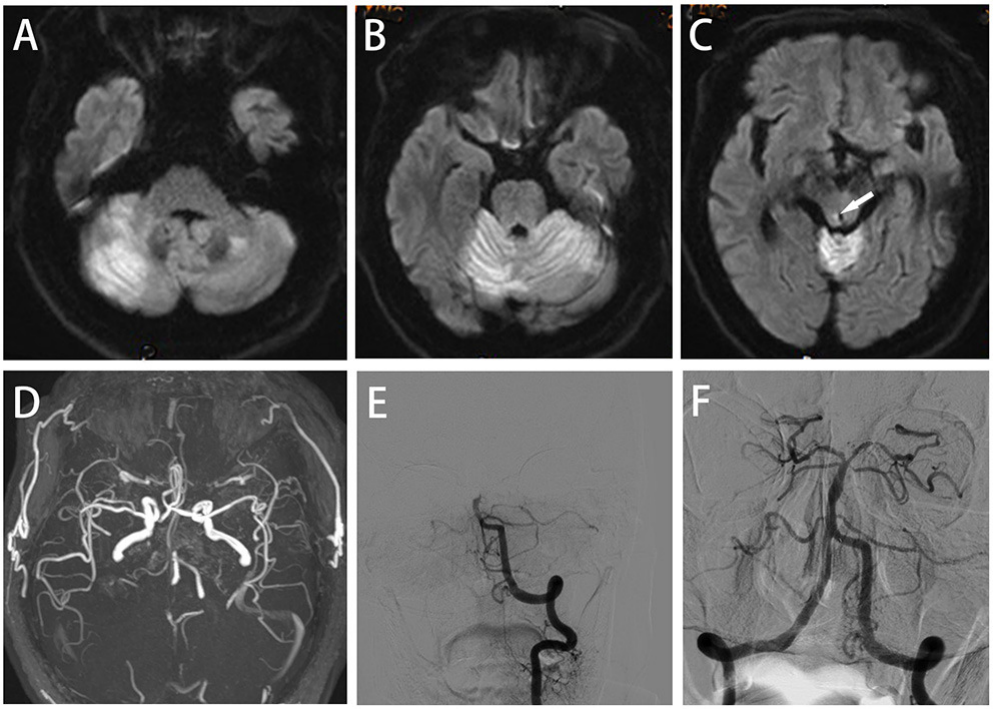
Case 3: **(A–C)** Axial diffusion-weighted imaging (DWI) showing acute cerebral infarction in the bilateral cerebellar hemispheres, cerebellar vermis and periaqueductal gray (arrowheads). **(D,E)** Magnetic resonance angiography (MRA) and AP left VA angiography demonstrating basilar artery occlusion; successful recanalization was obtained by endovascular thrombectomy **(F)**.

A 65-year-old male with a history of hypertension was admitted in the emergency department of our hospital with unconsciousness. Physical examination revealed excessive sweating, fever (40°C), tachycardia (120/min), tachypnea (23/min) and hypertension (180/105 mmHg). Neurological examination showed bilateral pupil diameter of 3.0 mm with disappearance of light reflex, deep coma, paroxysmal limb shaking, bilateral and spontaneous extensor posturing, loss of tendon reflex and bilateral positive Babinski's sign. His GCS score was 4. The above symptoms and signs were persistent except for limb shaking, which lasted for 10–15 s and occurred repeatedly at intervals of about 20–30 min without identifiable triggers. The diagnosis of PSH was made according to PSH-AM (PSH-AM score of 20). ECG showed atrial fibrillation. Epileptic discharges were not detected by EEG. No abnormal changes were found on CT scans of the head and chest. Laboratory examinations were normal. Brain MRI showed acute cerebral infarction in the bilateral cerebellar hemispheres, cerebellar vermis and periaqueductal gray (PAG) (Figures 3A–C). Magnetic resonance angiography (MRA) revealed basilar artery occlusion (Figure 3D). Subsequently, 1,000,000 IU urokinase were injected intravenously within 1 h, and the time from symptom onset to treatment was 5 h. However, the clinical symptoms of the patient were not significantly improved. DSA also confirmed the occlusion of the basilar artery (Figure 3E), which was recanalized after stent embolectomy (TICI grade III, Figure 3F). The time from symptom onset to recanalization was 480 min. The symptoms of paroxysmal limb shaking and muscular hypertonia of the extremities disappeared after surgery. The patient was still in a deep coma, febrile (42 °C), tachycardic (120/min), tachypneic (20/min) and hypertensive (168/100 mmHg) but did not reach the possible diagnosis of PSH. Subsequently, he was transferred to the NICU. He died of respiratory and circulatory failure only 10 h after the operation.

## Discussion

PSH mainly occurs in TBI, accounting for about 80% of all cases; other causes include anoxic brain injury (10%), stroke (5%) and rare causes (5%) (Meyfroidt et al., 2017). The prevalence of ischemic stroke with PSH is lower than that of hemorrhagic stroke (a ratio of 1:4) (Perkes et al., 2010). There are no clinical randomized controlled trials assessing PSH in ischemic stroke. However, a few cases of ischemic stroke with PSH have been reported. Deepika et al. reported a 6-year-old girl with Moyamoya disease who developed PSH on the 3^rd^ day after indirect revascularization surgery (Deepika et al., 2014). In addition, Verma et al. described a 42-year-old male patient with acute right middle cerebral artery occlusion who developed PSH symptoms on the 10^th^ day of onset (Verma et al., 2015). Similarly, Siefferman et al. reported a 41-year-old male patient who developed PSH on the 8^th^ day after acute right middle cerebral artery ischemic stroke (Siefferman and Lai, 2015). Furthermore, Godoy et al. reported two young male patients with PSH symptoms after cerebral fat embolism (Godoy et al., 2018). Recently, Surathi et al. described a 64-year-old male patient who developed PSH symptoms 3 months after pontine infarction (Surathi et al., 2021). The patients reported in the above cases had better response to the treatment. Here, PSH as the first manifestation of acute LVO in the posterior circulation was reported in all three cases. To our knowledge, there is no related case report at present.

There was no unified diagnostic standard until the international consensus was reached in 2014 when the term of PSH was introduced (Baguley et al., 2014). The PSH-AM was designed to assess patients continuously from admission to intensive care unit (Baguley et al., 2014). The scale consists of two separate parts, including the clinical feature scale (CFS) and diagnosis likelihood tool (DLT) scores. The sum of CFS and DLT scores was used to evaluate the reliability of PSH diagnosis, with < 8 as unlikely, 8–16 as possible, and ≥17 as probable. All three patients we reported were assessed before treatment and the PSH-AM scores were >17, indicating probable diagnoses. However, in addition to rhythmic limb shaking, the clinical feature of paroxysmal limb shaking was not observed in these three patients. This may be explained by the following reasons: Firstly, different from TBI, acute ischemic stroke with PSH as the first manifestation can be treated by thrombolysis or mechanical thrombectomy. Secondly, the time from PSH symptom onset to the recovery of brain tissue reperfusion is too short to observe the frequency of PSH signs.

PSH occurs at any stage during the disorder that causes it (Godoy et al., 2019). However, it is usually observed in the first week after TBI. In the early stages of TBI, patients are often sedated to avoid secondary brain injury. This may mask the early classic features of PSH and delay diagnosis. Therefore, PSH as the first clinical manifestation is extremely rare, e.g., the three patients reported in this study. Previous studies have shown that chronic occlusion of the basilar artery caused by atherosclerosis often leads to small infarct lesions or transient ischemic symptoms. However, sudden embolism often causes extensive ischemic damage and poor clinical prognosis (Mattle et al., 2011). Notably, all the three current patients were complicated with atrial fibrillation. We speculate that embolism may be involved in the occurrence of PSH symptoms.

At present, which site of brain damage increases the odds of developing PSH remains unestablished. In 2015, Hinson et al. showed that disconnection between the posterior corpus callosum and the posterior limb of the internal capsule may promote the development of PSH (Hinson et al., 2015). LV et al. found that involvement of the corpus callosum, deep gray nuclei, periventricular white matter and midbrain/pons can increase the odds of developing PSH (Lv et al., 2010). Multiple infarct lesions were distributed in posterior circulation in our three patients. In case 1, cerebral infarction lesions were widely distributed in the posterior circulation, involving the corpus callosum, thalamus, temporal lobe, occipital lobe, cerebellum, midbrain and pons. Like case 1, case 2 also involved these structures, except the temporal lobe and corpus callosum, corroborating LV et al.'s study. Brain MRI showed that the lesion in case 3 involved the cerebellar hemisphere and PAG. We speculate that extensive disconnection due to multiple lesions in posterior circulation may play an important role in the occurrence and development of PSH. Coincidentally, a common feature of the three cases was that that the infarcted lesions involved the midbrain. A literature review showed that the PAG is presumed to play an important role in the central inhibitory driver, and structural or functional impairment of the midbrain may underlie the severer end of the PSH spectrum (Tang et al., 2009). This notion can also explain why the three patients still had a poor prognosis despite early endovascular treatment.

Currently, the evidence level of the best treatment strategy for PSH is low, being limited to case reports or case series. In practice, drug combinations with different mechanisms of action are used to control and prevent PSH symptoms. Meyfroidt et al. proposed three main treatment objectives for patients with PSH, including refraining from triggering factors, reducing excessive sympathetic outflow and nutritional support therapy (Meyfroidt et al., 2017). However, treatment of the primary disease causing PSH may play a crucial role. In the current three patients with acute LVO in the posterior circulation, PSH symptoms did not recur after the occluded vessels were recanalized by endovascular treatment.

## Conclusion

PSH can be the initial symptom of acute LVO in the posterior circulation. Extensive disconnection due to multiple lesions in posterior circulation may promote the occurrence and development of PSH. Endovascular therapy may be effective for PSH signs caused by acute posterior circulation-related ischemic stroke. Further research is needed in the future for verification.

## Data Availability Statement

The original contributions presented in the study are included in the article/supplementary material, further inquiries can be directed to the corresponding author.

## Ethics Statement

The studies involving human participants were reviewed and approved by the Ethics Committee of Xingtai Third Hospital (approval number: 2021-KY-28). The patients/participants provided their written informed consent to participate in this study. Written informed consent was obtained from the individual(s) for the publication of any potentially identifiable images or data included in this article.

## Author Contributions

All authors listed have made a substantial, direct, and intellectual contribution to the work and approved it for publication.

## Funding

This study was supported by the Projects in Science and Technique Plans of Xingtai City [grant number 2021ZC108].

## Conflict of Interest

The authors declare that the research was conducted in the absence of any commercial or financial relationships that could be construed as a potential conflict of interest.

## Publisher's Note

All claims expressed in this article are solely those of the authors and do not necessarily represent those of their affiliated organizations, or those of the publisher, the editors and the reviewers. Any product that may be evaluated in this article, or claim that may be made by its manufacturer, is not guaranteed or endorsed by the publisher.
